# Differential Migration and Activation Profile of Monocytes after Trophoblast Interaction

**DOI:** 10.1371/journal.pone.0097147

**Published:** 2014-05-21

**Authors:** Esteban Grasso, Daniel Paparini, Vanesa Hauk, Gabriela Salamone, Claudia Perez Leiros, Rosanna Ramhorst

**Affiliations:** 1 Immunopharmacology Laboratory, School of Sciences, University of Buenos Aires and IQUIBICEN- CONICET (National Research Council), Buenos Aires, Argentina; 2 Immunology Department, Instituto de Investigaciones Hematológicas e Instituto de Estudios Oncológicos “Fundación Maissa”; Academia Nacional de Medicina, Buenos Aires, Argentina; Queen’s University, Canada

## Abstract

Macrophages at the maternal-placental interface coordinate opposite demands under the control of trophoblast cells such as the response against pathogens on one hand, and apoptotic cell clearance and wound healing with the production of suppressor cytokines. Here, we investigated whether trophoblast cells induce maternal monocyte activation towards an alternative activated macrophage profile and whether bacterial or viral stimuli modulate their migratory properties. We used an *in vitro* model of the maternal-placental interface represented by co-cultures of CD14+ cells isolated from fertile women with first trimester trophoblast cell line (Swan-71 cells) in the presence or absence of pathogen associated molecular pattern (PAMP) stimuli lipopolysaccharide (LPS), peptidoglycan (PGN) or poly [I:C]). Maternal CD14+ cells showed increased CD16 and CD39 expression, both markers associated to an alternative activation profile, with no changes in CD80 expression after trophoblast cell interaction. These changes were accompanied by increased IL-10 and decreased IL-12 production by CD14+ cells. After stimulation with LPS, PGN or poly [I:C], monocytes co-cultured with trophoblast cells had lower production of TNF-α and IL-1β compared with non co-cultured monocytes. Interestingly, monocyte migration towards trophoblast cells was prevented in the presence of LPS or PGN but not after 24h of stimulation with poly [I:C]. LPS or PGN also decreased CCR5, CXCL-8 and CCL5 expression. Finally, trophoblast cells co-cultured with monocytes in the presence of pathological stimuli failed to increase chemokine expression, indicating a bidirectional effect. In conclusion, trophoblast might ‘instruct’ maternal monocytes to express an alternative activation profile and restrain their early recruitment under pathological threats as one of the first strategies to avoid potential tissue damage at the maternal-placental interface.

## Introduction

The control of immune homeostasis at the maternal-placental interface involves several and redundant immunoregulatory circuits. From an immunological standpoint, pregnancy evolves through different stages with predominant pro-inflammatory or anti-inflammatory profiles depending on the stage of gestation [1], [2]. Trophoblast invasion, tissue remodelling and angiogenesis thus occur under a regulated microenvironment [3]–[6] that involves active immunosuppressant and tolerogenic circuits such as the selective recruitment of non-cytotoxic NK CD16^-^CD56^bright^ cells that synthesize angiogenic and growth factors, the induction of regulatory T cells (Treg) and expansion of natural Tregs, the induction of tolerogenic dendritic cell profile and decidual macrophage differentiation to alternative activated phenotypes, among others [7]–[12]. Particularly, macrophages represent one of the major leukocyte subsets in decidua throughout pregnancy [13], [14]. During early normal pregnancy, macrophages bear a predominant alternative activation profile contributing to suppressor cytokine and wound healing mediator synthesis. However, macrophages can express a classical inflammatory profile to control the risk of infection by ascending or blood-borne pathogens [13].

In this sense, evidence indicates that macrophage functional profiles are determined by the kind of stimulus and the specific micro-environmental conditions in which cells were differentiated prior to their activation [14], [15]. Fest et al. have previously shown that trophoblast cells secrete chemokines able to recruit maternal macrophages and to modify their secreted cytokine profile [16]. The selective recruitment of different leukocyte populations through a chemokine network also constitutes an additional checkpoint for homeostasis maintenance at the early maternal-placental interface, even in the presence of threatened infection [17]–[19]. In fact, chemokines are central to innate and adaptive immunity and they control physiological processes such as wound healing and angiogenesis as well as embryo growth and development [20], [21]. Trophoblast cells actively recruit immune cells through chemokine production [1], [22], [23] and they can also affect immune cell function following the recognition of pathogen associated molecular patterns (PAMPs) expressed on bacteria, virus, parasite and fungi through toll like receptors (TLR) [3], [24]–[26]. Stimulation of human trophoblast cells through TLR4 by lipopolysaccharide (LPS), TLR2 by peptidoglycan (PGN) or TLR3 by polyinosinic:polycytidylic acid (poly [I:C]) (a synthetic analogue of viral dsRNA) increases the production of inflammatory chemokines with strong chemottractant effect on CD14+ monocytes to the site of implantation [25], [27]. Accordingly, a deregulated inflammatory response during implantation with enhanced leukocyte infiltration may be an underlying cause of pregnancy complications [13], [19].

On the basis that trophoblast cells contribute to maternal monocyte differentiation to macrophage alternative activation profiles, we hypothesized that trophoblasts under pathogen stimulation modulate chemokine networks that act on monocytes/macrophages as a strategy to avoid potential tissue damage and pregnancy loss. In the present work, we showed that trophoblast cells, in the presence of stimuli mimicking bacterial or viral infections, differentially induce the activation of maternal monocytes to alternative activated macrophage profile and modulated chemokine and chemokine-receptor expression affecting their migratory properties.

## Materials and Methods

### Blood Samples

Blood samples were obtained from fertile women, defined as women who had two or more previous normal pregnancies without any miscarriage in their clinical record, non-smokers, and under no pharmacological treatment for at least 10 days before the sampling day (mean age 33,2 years range 26–42 years, n = 12). Blood was obtained by puncture of the forearm vein, and it was drawn directly into heparinized plastic tubes. These studies were approved by the “Academia Nacional de Medicina Review Board” and Ethical Committee. All healthy donors provided written informed consent for sample collection and subsequent analysis.

### Monocyte Isolation

Peripheral blood mononuclear cells (PBMC) from fertile women were isolated by density gradient centrifugation on Ficoll-Hypaque (Amersham Pharmacia Biotech, Uppsala, Sweden). CD14+ cells were separated by positive selection with CD14+ micro magnetic beads according to the manufacturer’s instructions (Miltenyi Biotec., Bergisch Gladbach, Germany). Cell population purity was checked by FACS analysis using anti-CD14 mAb and was found to be >95% for each set of experiments.

### Trophoblast Cells Cultures

Trophoblast cells (Swan-71 cell line, derived by telomerase-mediated transformation of a 7-weeks cytotrophoblast isolate described by Straszewski-Chavez) [28], were cultured in 24-well flat bottom polystyrene plates in complete DMEM 10% FCS (Gibco, Invitrogen, Life Technologies, Grand Island, NY, USA).

For co-cultures, trophoblast cells at 70% confluence (2×10^5^ cells/well) were cultured in the absence/presence of CD14+ (2.5×10^5^ cells/well) cells either in direct contact co-culture or using a non-migration 0.4 µm transwell system. After 24 hours of culture, supernatants were collected for ELISA determinations and cells were recovered for FACS or RT-PCR analysis.

### Flow Cytometry Analysis

Maternal monocytes were directly co-cultured with Swan-71 cells and after 24 hours cells were recovered by TrypLe treatment (Invitrogen Life Technologies, Grand Island, NY, USA) and stained with the following monoclonal Abs: mAb anti CD14 (PE-conjugated), CD16 (FITC-conjugated), CD39 (APC-conjugated), and CD80 (PE-Cy5.5-conjugated) (BD Biosciences, San Jose, CA) for surface staining. For intracellular cytokine detection Stop Golgi was added to the medium in the last 4 hours of co-culture following manufacturer’s instructions (Becton Dickinson, San José, CA), to promote intracellular accumulation. Then cells were recovered and, after washing with PBS-2%FCS, cells were stained with mAb anti CD14 (FITC-conjugated) (Becton Dickinson, San José, CA), then washed, fixed and permeabilized with the Fix/Perm kit as manufacturer recommended (Becton Dickinson, San José, CA). Permeabilized cells were incubated for 30 min with PE-conjugated anti IL-10 mAb or IL-12 mAb (PE-conjugated) (BD Biosciences, San Jose, CA). Cells were finally washed with PBS-2% FCS.

Twenty thousand events were acquired in a FACSAria II cytometer and results were analyzed using Cyflogic1.2.1 Software. Negative control samples were incubated in parallel with an irrelevant, isotype-matched Ab. Results are expressed as fold increase of the mean fluorescence intensity (MFI) of the marker of interest relative to negative MFI control.

### Migration Assays

We evaluated the migration of maternal monocytes towards Swan-71 cells using 8 µm transwell systems (BD Falcon cell culture inserts). Isolated CD14+ cells were seeded in the upper compartment (2.5×10^5^ cells/insert) and trophoblast condition media in the lower compartment as attractant. Trophoblast conditioned media were obtained from trophoblast cells cultured in complete DMEM 2% FCS with different PAMP stimuli representing a bacterial or viral infection: LPS (lipopolysacharide 10 µg/ml isolated from Escherichia coli (0111:B4)), PGN (peptidoglycan purified from Gram-positive bacteria, 10 µg/ml), or poly [I:C], (polyinosinic:polycytidylic acid, synthetic analog dsRNA, 10 µg/ml) all from (Sigma–Aldrich, St Louis, MO, USA) for 24 hours. After 24 and 48 hours, the cells were recovered from the lower and upper compartments and the frequency of CD14+ cells were quantified by FACS analysis. DMEM 20% human serum was used as a positive migration control. Results are expressed as percentage of migration: CD14+ cells in the lower compartment/total CD14+ cells *100. For these experiments we considered the spontaneous migration as the % of CD14 cells recovered from the lower compartment without attraction stimuli.

To evaluate RANTES-contribution to the recruitment of maternal CD14+ cells by trophoblast cells under poly [I:C] stimulation, we performed the migration assay pre-incubating condition media or not with anti-RANTES neutralizing Ab (10 µg/ml, R&D System MN, USA). These results are expressed as fold increase of migration relative to basal conditioned media.

### Chemokines, Chemokine Receptors and TLR Expression

We evaluated chemokines, their receptors and TLR expression in maternal monocytes and in Swan-71 cells before and after bidirectional interaction. Trophoblast cells were cultured in a 24-well flat bottom polystyrene plate in complete DMEM 10% FCS. When the cells reached 70% confluence LPS (10 µg/ml), PGN (10 µg/ml) or poly [I:C] (10 µg/ml) were added and then 0.4 µm inserts containing isolated monocytes (2.5×10^5^ cells/insert) (BD Falcon cell culture inserts) were fitted. After 24 hours, supernatants were collected for IL-1β and TNF-α determination, monocytes recovered from the upper compartment and trophoblast cells from the lower compartment to determine chemokine and chemokine receptor expression by RT-PCR. Briefly, total RNA was isolated following manufacturer recommendations with Trizol reagent (Life Technologies, Grand Island, NY, USA), cDNAs were generated from 1 µg or RNA using a MMLV reverse transcriptase, RNAsin RNAse inhibitor and oligodT kit (Promega Corporation, Madison, WI, USA) and stored at −20°C for batch analysis. The sample volume was increased to 25 µl with the solution containing 50 mM KCl; 10 mM Tris (pH 8.3); 1.5 mM MgCl_2_; 0.1 µM forward and reverse primers (described in table 1) and 1 U Taq polymerase in a DNA Thermocycler (PerkinElmer/Cetus, Boston, MA, USA). The primers and the thermal profile were selected with PrimerBlast software (www.ncbi.nlm.nih.gov/tools/primer-blast/). The corresponding PCR programs are described in Table 2. PCR products were electrophoresed through a 2% ethidium bromide-stained agarose gel, visualized by transillumination and scanned. Densitometry was performed using ImageJ 1.47 software (NIH, USA) and results expressed as arbitrary units normalized to GAPDH expression.

**Table 1:** Sequence of the primers used.

Primers	Sequence	Product (bp)
**GAPDH**	*sense* 5′ – TGATGACATCAAGAAGGTGGTGAAG –3′	240
	*antisense* 5′ – TCCTTGGAGGCCATGTAGGCCAT –3′	
**CCR1**	*sense* 5′ – GCCTGAAACAGCTTCC –3′	527
	*antisense* 5′ – AGAAGGTGAACGAGAGG –3′	
**CCR3**	*sense* 5′ – GTTGGTCATAATTCGGAGCCTCC –3′	134
	*antisense* 5′ – AAAGCTGATACCAGAGCACTGATGG –3′	
**CCR5**	*sense* 5′ – TTCTGAACTTCTCCCCGACAAA –3′	280
	antisense 5′ – TGCTACTCGGGAATCATAAAAACT –3′	
**CCL5 (RANTES)**	*sense* 5′ – TGCTGCTTTGCCTACATTGC –3′	95
	antisense 5′ – AAGACGACTGCTGGGTTGG –3′	
**CXCL8 (IL-8)**	*sense* 5′ – CCAACACAGAAATTATTGTAAAGC –3′	163
	antisense 5′ – CACTGGCATCTTCACTGATTC –3′	
**CCL2 (MCP1)**	*sense* 5′ – CAGCAGCAAGTGTCCCAAAG –3′	146
	*antisense* 5′ – GAGTGAGTGTTCAAGTCTTCGG –3′	
**TLR2**	*sense* 5′ – GAGTTCTCCCAGTGTTTG –3′	162
	*antisense* 5′ – CATTGTCCAGTGCTTCA –3′	
**TLR3**	*sense* 5′ – GTGCCGTCTATTTGCCA –3′	102
	*antisense* 5′ – AGTCTGTCTCATGATTCTGTTG –3′	
**TLR4**	*sense* 5′ – CGTGAGACCAGAAAGCTG –3′	157
	*antisense* 5′ – ATAGCTGCCTAAATGCCTC –3′	

**Table 2:** Description of the PCR amplification programs.

	CCL5, CCL2, CXCL8, GapDH	CCR1 CCR3, CCR5	TLR2, TLR3, TLR4
Initial denaturalization	**95**°**C, 5 min**	**95**°**C, 5** **min**	**95**°**C, 5 min**
Cycles	**35**	**35**	**35**
- Denaturalization	**95**°**C, 20s**	**95**°**C, 1 min**	**95**°**C, 20s**
- Primer annealing	**62**°**C, 20s**	**55**°**C, 1 min**	**55**°**C, 20s**
- Elongation	**72**°**C, 20s**	**72**°**C, 1 min**	**72**°**C, 20s**
Final elongation	**72**°**C, 10 min**	**72**°**C, 15 min**	**72**°**C, 10 min**

### Measurement of Cytokine Production by ELISA

Swan-71 cell line at 70% confluence (2×10^5^ cells/well) and purified CD14+ cells (2.5×10^5^ cells/well) were cultured in a 24-well polystyrene plate in complete DMEM 10% FCS stimulated or not with LPS (10 µg/ml), PGN (10 µg/ml), or poly [I:C] (10 µg/ml). Alternatively, Swan-71 cells and CD14+ cells were co-cultured using 0.4 µm pore transwell system as previously described with the different stimuli. After 24 hours, supernatants were collected, centrifuged, and analyzed for the presence of IL-1β and TNF-α by ELISA (R&D System, MN, USA).

### Statistical Analysis

Statistical significance was determined using Wilcoxon test, a non-parametric paired t-test, for comparing monocytes with or without co-culture. For those assays that involved multiple stimuli comparisons to the basal condition, we used the Friedman test, a non-parametric matched ANOVA, followed by Dunn’s post-test. Statistical analysis was performed using GraphPad Prism5 (GraphPad, San Diego, CA, USA) and p-value<0.05 was considered significant.

## Results

### Trophoblast Cells Condition Maternal CD14+ Cell Profile

**Figure 1:**
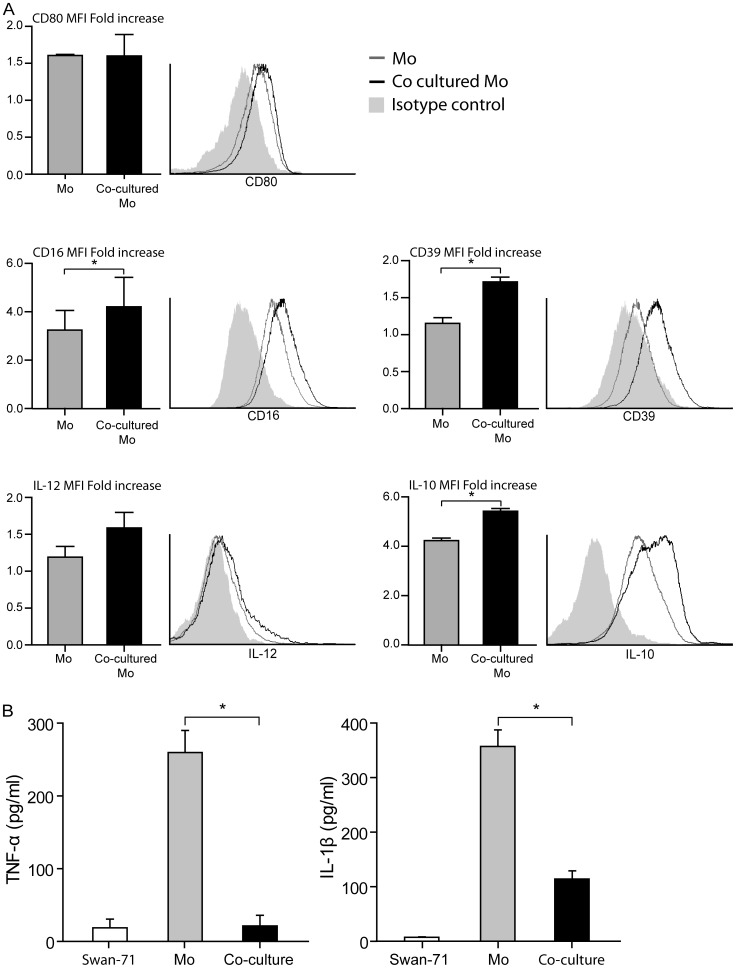
Trophoblast cells induce and alternative profile on maternal CD14+ cells. Maternal monocytes purified by magnetic beads were cultured or not with Swan-71 cells at 70% of confluence in a 24 well flat-bottom plate. After 24 hours of culture, cells were recovered by TrypLE treatment and CD80, CD16, CD39, IL-12 and IL-10 were quantified by FACS on CD14+ gate (**A**). Results are expressed as fold increase of the MFI of the marker of interest relative to its isotype control (mean ± SEM) (*p<0.05, Wilcoxon test). Figure also shows a representative histogram of 3 independent experiments. At the same time, supernatants were recovered and TNF-α and IL-1β were quantified by ELISA (**B**). Results are expressed as mean ± SEM pg/ml from 3 independent experiments (*p<0.05, Wilcoxon test).

We first explored the maternal CD14+ cell profile after interaction with trophoblast cell line Swan-71. Figure 1A shows no modulation of co-stimulatory molecule CD80, characteristic marker of pro-inflammatory stimulation, on monocytes that have interacted with trophoblast cells (educated monocytes). On the other hand, trophoblast cell co-culture significantly increased CD14+CD16+ and CD14+CD39+ expression. CD16 is the FcγRIII, a marker associated with a regulatory macrophage profile [14] and CD39 is a surface enzyme that was proposed as self-limiting for macrophage pro-inflammatory activation based in its rapid catabolism of endogenous ATP into adenosine [24]. This phenotype profile was accompanied by changes in CD14+ cell functional profile with increased IL-10 synthesis but not of IL-12 after the interaction with Swan-71 cells (Figure 1A). IL-1β and TNF-α were then quantified in supernatants collected after 24 hours of co-culture between trophoblast cells and monocytes. Figure 1B shows a significant reduction in the secretion of both inflammatory cytokines.

### Trophoblast Cells Prevent Inflammatory Cytokine Release by CD14+ Cells Under PAMP Stimulation

**Figure 2:**
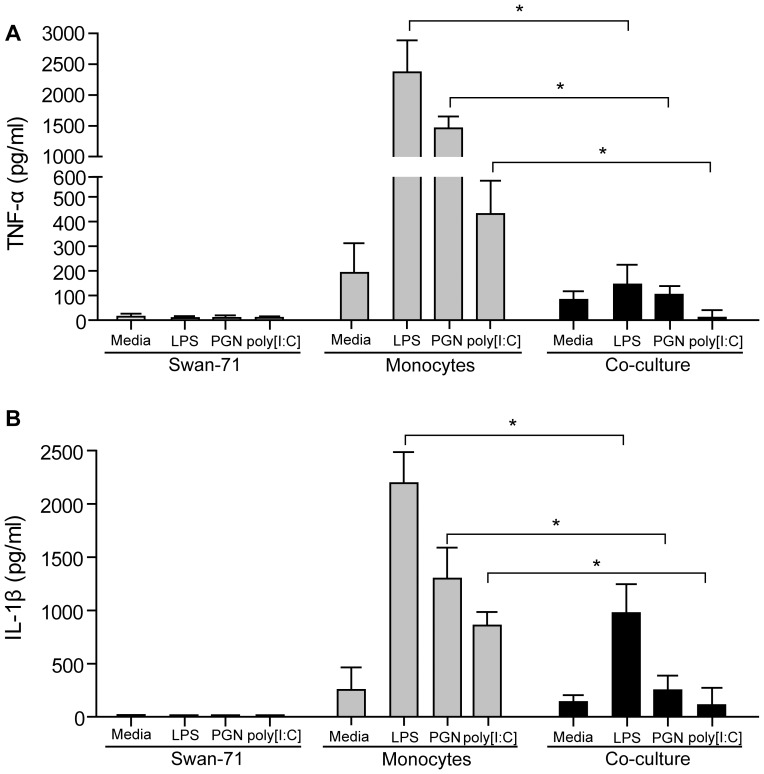
Trophoblast cells prevent inflammatory cytokine release by CD14+ cells under PAMP stimulation. Maternal monocytes were co-cultured or not with Swan-71 cells in the absence or presence of LPS (10 µg/ml), PGN (10 µg/ml), or poly [I:C] (10 µg/ml). After 24 hours supernatants were collected to quantify TNF-α (**A**) and IL-1β (**B**) by ELISA. Results are expressed as mean ± SEM pg/ml from 3 independent experiments (*p<0.05 Wilcoxon test).

Since trophoblast interaction promoted the expression of an alternative activation profile in CD14+ cells, we next investigated the effect of PAMP stimuli on their cytokine secretion profile. Swan-71 cells were cultured in the absence or presence of LPS, PGN or poly [I:C] resembling bacterial and viral stimulation in the lower compartment, and purified monocytes were added to the upper compartment. After 24 hours supernatants were collected and IL-1β and TNF-α production quantified by ELISA. As shown in Figures 2A and 2B, significantly lower production of TNF-α and IL-1β was produced by CD14+ cells under trophoblast conditioning in comparison with non co-cultured cells, suggesting that trophoblast cells can regulate pro-inflammatory cytokine production at early stages of stimulation.

### Trophoblast Cells Restrain Early Monocyte Migration Depending on the Type of PAMP Stimulus

**Figure 3:**
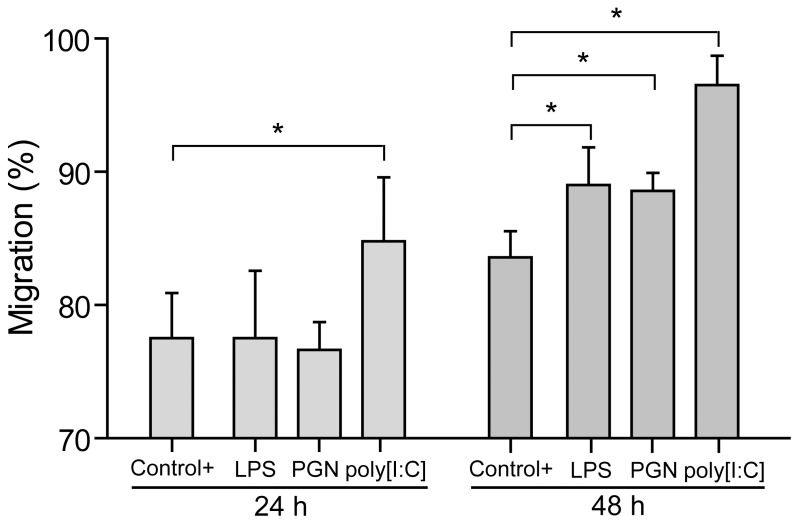
Trophoblast cells restrain maternal monocytes migration depending on the pathological stimuli. Migration assays were performed in transwell system. Condition media from Swan-71 cells cultured in the absence or presence of LPS (10 µg/ml), PGN (10 µg/ml), or poly [I:C] (10 µg/ml) was used in the lower compartment as attractant and 8 µm-pore inserts were added containing the isolated monocytes (upper compartment). After 24 and 48 hours, cells were recovered from the lower and upper compartments and the frequency of CD14+ cells were quantified by FACS analysis. As a positive control, we used 20% human AB serum. Results are expressed as percentage of migration (mean ± SEM) from 4 migration assays using different fertile women (*p<0.05, Friedman test followed by Dunn’s post-test).

Taking into account that trophoblast cells produce several chemokines that selectively recruit maternal monocytes toward the maternal-placental interface [16], [29], we wondered whether trophoblast cells primed with bacterial or viral PAMP stimulation could modify the early recruitment of maternal monocytes. Therefore, we performed migration assays of isolated monocytes, seeded on a 8 µm-pore insert (upper compartment), toward condition media of trophoblast cells cultured in the absence or presence of LPS (10 µg/ml), PGN (10 µg/ml) or poly [I:C] (10 µg/ml) (lower compartment). After 24 and 48 hours, we quantified CD14+ cells in the upper and lower compartment by FACS analysis. As it is depicted in Figure 3, after 24 hours only poly [I:C] induced CD14+ cell migration whereas LPS or PGN did not increase migration toward Swan-71 cell medium. However, at 48 hours all three PAMP stimuli increased CD14+ cell migration suggesting differential stimulus-dependent migratory properties of monocytes at the very first stages of trophoblast interaction.

### Trophoblast Cells Condition Chemokine and Chemokine Receptor Expression on Monocytes Under PAMP Stimulation

**Figure 4:**
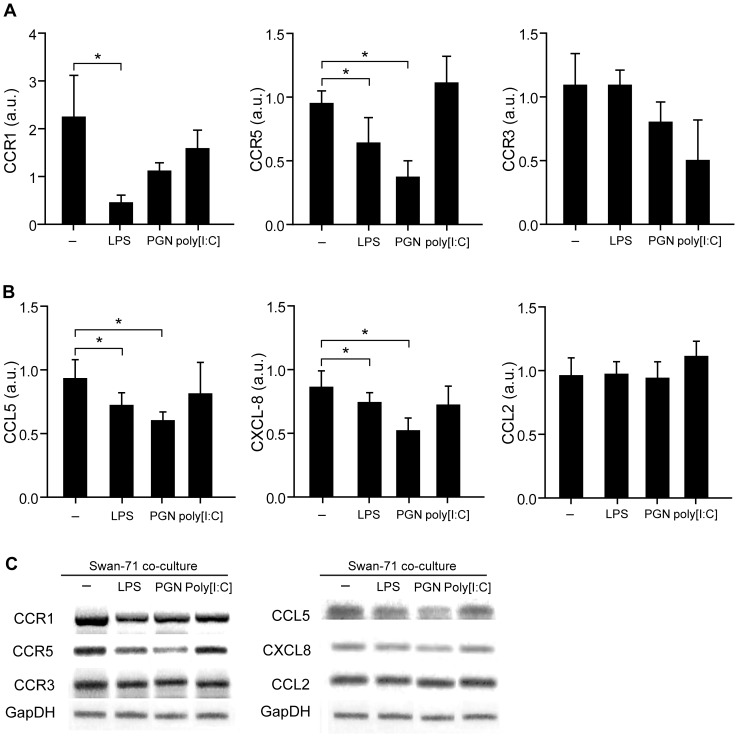
Trophoblast cells primed with PAMPs stimuli condition chemokine and chemokine receptor expression on maternal monocytes. Swan 71 cell line at 70% of confluence were cultured in a polystyrene plate in complete DMEM 10% FCS in the absence or presence of LPS (10 µg/ml), PGN (10 µg/ml), or poly [I:C] (10 µg/ml) and 0.4 um pore-insert were added containing CD14+ cells. After 24 hours, cells were recovered from the upper compartments and the expression of (**A**) CCR1, CCR5 and CCR3; (**B**) CCL5, CXCL8, and CCL2 were evaluated by RT-PCR and normalized to GAPDH expression. Results are expressed as arbitrary units (a.u) (mean ± SEM) from 6 independent experiments using different fertile women (*p<0.05 Friedman test followed by Dunn’s post-test). (**C**) Representative amplification bands from 6 independent experiments.

Since monocytes showed a restricted migration toward trophoblast cells in the presence of LPS or PGN after 24 hours of stimulation, CD14+ cells were analyzed to determine chemokine and chemokine receptor expression known to have a role in the generation of the maternal-placental interface [26]. For these sets of experiments we performed co-cultures in transwells with 0.4 µm pore to avoid direct cellular contact and to permit us to work with isolated populations. Monocytes were seeded on the upper compartment and cultured in the absence or presence of Swan-71 cells with LPS, PGN or poly [I:C] (lower compartment). After 24 hours of culture, CCR1, CCR3 and CCR5 expression was evaluated by RT-PCR in monocytes recovered from the upper compartment. As depicted in Figure 4A, CCR1 and CCR5 expression significantly decreased in the presence of LPS, and CCR5 also decreased in the presence of PGN. Interestingly, cell interaction could not modulate poly [I:C] effect on CCR5 and CCR1. No modulation of CCR3 expression was observed with any of the PAMP stimuli tested.

On the other hand, chemokine expression in monocytes recovered after culture with Swan-71 cells and PAMPs stimuli was assessed, particularly focusing on CCL2 (MCP-1), CCL5 (RANTES) and CXCL8 (IL-8) due to their ability to regulate macrophage chemotaxis within the decidua. Figure 4B shows a significant reduction of CCL5 and CXCL8 in CD14+ cells cultured with LPS and PGN but not in the presence of poly [I:C]. In addition, we did not observe modulation in CCL2 expression under basal conditions or after pathogenic stimuli. Figure 4C shows representative amplification bands of CCR1, CCR3, CCR5, CCL2, CCL5 and CXCL8 in monocytes after cultured with trophoblast cells and PAMPs stimulation.

### Trophoblast Cells Selectively Reduce Chemokine Expression after the Interaction with CD14+ Cells

**Figure 5:**
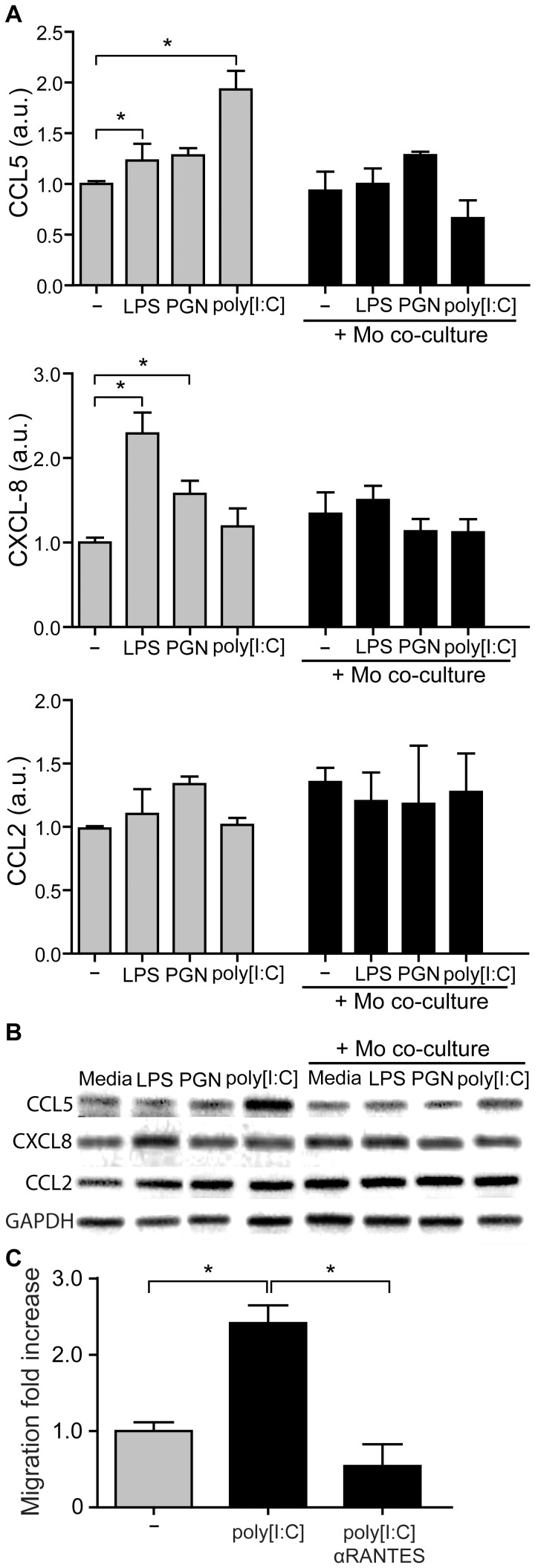
Trophoblast cells selectively reduce chemokine production after the interaction with maternal monocytes in the presence of PAMPs stimuli. Swan-71 cell line was cultured with LPS (10 µg/ml), PGN (10 µg/ml), or poly [I:C] (10 µg/ml) in a polystyrene plate in the absence or presence of 0.4 um pore-insert with CD14+ cells. After 24 hours, Swan-71 cells were recovered from the lower compartments and the expression of CCL5, CXCL8 and CCL2 (**A**) was evaluated by RT-PCR and normalized to GAPDH expression. Results are expressed as arbitrary units (a.u) (mean ± SEM) from 3 independent experiments using different fertile women (*p<0.05, Friedman test followed by Dunn’s post-test). (**B**) Representative amplification bands from 3 independent experiments. (**C**) Migration assays were performed in transwell system. Condition media from Swan-71 cell cultured in the absence or presence of poly [I:C] (10 µg/ml) was used as attractant in the lower compartment pre-treated or not with anti-CCL5 neutralizing Ab and 8 µm-pore inserts containing the isolated monocytes were added (upper compartment). After 24 hours, cells were recovered from the lower and upper compartments and the frequency of CD14+ cells were quantified by FACS analysis. Results are expressed as fold increase of CD14+ migration relative to basal condition media migration (mean ± SEM) from 3 independent migration assays (*p<0.05, Friedman test followed by Dunn’s post-test).

Taking into account that homeostasis maintenance at the maternal-placental interface requires continuous cross-talk between trophoblast cells and maternal leukocytes, our next step was to evaluate the ability of Swan-71 cells to modulate their own chemokine expression pattern in a pathological stimulus-induced microenviroment and the effect of a previous interaction with maternal monocytes. For that purpose transwell assays were performed with Swan-71 cells stimulated or not with PAMPs in the lower compartment and CD14+ cells in the upper compartment. After 24 hours, trophoblast cells were recovered and chemokine expression evaluated by RT-PCR. Figure 5A shows higher expression of CCL5 and CXCL8 under certain PAMP stimuli in Swan-71 cells that had not interacted with monocytes. However, after interacting with maternal monocytes, trophoblast cells did not modulate the expression of these chemokines by PAMP stimulation. Particularly, after trophoblast-monocyte interaction, the poly [I:C] effect on CCL5, and LPS and PGN effects on CXCL8 were prevented (Figure 5A). Figure 5B shows representative amplification bands of CCL2, CCL5 and CXCL8 in Swan-71 under PAMPs stimulation cultured in the absence or presence of 0.4 um pore-insert with CD14+ cells.

Since PAMPs sitimuli could also modulate TLR expression on trophoblast cells we evaluated TLR2, TLR3 and TLR4 expression following the LPS, PGN or poly [I:C] exposure. There was no effect on TLR expression after 24 hours of PAMPs sitmuli determined by RT-PCR (data not shown).

Based on the observations that CD14+ cell migration appeared not restricted after 24 hours of co-culture with trophoblast cells and that trophoblast cells, in turn, significantly increased CCL5 production after poly [I:C] stimulation, we next evaluated the contribution of CCL5 to CD14+ cell migration toward Swan-71 cells. Thus, migration assays were carried out in the absence/presence of anti-CCL5 neutralizing Ab. As shown in Figure 5C anti-CCL5 Ab decreased CD14+ cell migration toward Swan-71 cells stimulated with poly [I:C].

## Discussion

Trophoblast cells coordinate key cellular processes by the selective recruitment of leukocytes to the maternal-placental interface and produce soluble and contact factors that contribute to the generation of a tolerogenic microenvironment for homeostasis maintenance [30]–[32].

Results presented herein provide experimental evidence that trophoblast can regulate monocyte migration and activation through the regulation of chemokine network expression. Our conclusion is based on several observations. First, maternal monocytes after 24 hours of interaction with Swan-71 cells increase CD16 and CD39 expression, markers associated with immunoregulatory properties on CD14+ cells and activation in an alternative profile [14], [15], [29], [33]. These changes were accompanied by an increase in the production of IL-10 and decreased pro-inflammatory cytokine production. Second, LPS and PGN treatment failed to promote maternal monocyte migration at 24 hours in those co-cultures performed with trophoblast cells, however this effect was not present at 48 hours of culture suggesting an early temporal regulation. Third, the changes observed in monocyte migration properties with LPS or PGN were accompanied by a decreased expression of chemokines such as CXCL8 and CCL5 and chemokine receptors CCR1 and CCR5.

Macrophages bearing a predominant alternative activation profile are involved in the human placentation process and the transition of the pro-inflammatory response characteristic of implantation to an immunosuppressive profile in the second trimester [32], [34], [35]. In this study, we demonstrated that CD16 which represent a monocyte subpopulation with a unique functional role to regulate inflammation [34], [36] was up-regulated. In line with this, we showed that maternal CD14+ cells increased CD39 after the interaction with trophoblast cells, a surface enzyme that self-limits the activation process [33]. In this sense, recently Cohen et al. have demonstrated that TLR-stimulated macrophages increase adenosine triphosphate (ATP) synthesis and secretion as well as its rapid catabolism to adenosine by CD39, inducing a transition to a regulatory state. Therefore, CD39 would act as a key “molecular switch” that allows macrophages to self-limit their activation [33].

On the other hand, we investigated CCL2, CCL5 and CXCL8 expression based on their synthesis by endometrial and trophoblast cells and their role in physiologic and pathologic pregnancies [37], [38]. Particularly, CCL5 also called RANTES (Regulated on activation, normal T cell expressed and secreted) promotes an adequate pro-implantatory microenvironment that influences trophoblast cell survival and modulates the balance of maternal regulatory and effector T lymphocytes in favor of maternal tolerance; and CXCL8 (IL-8) is also produced by trophoblast cells associated with the invasion process [19], [31], [37]. LPS and PGN reduced chemokine production and also chemokine receptor expression on educated maternal monocytes regulated by trophoblast interaction making them ‘less sensitive’ to be recruited toward trophoblast cells. This could serve as a potential strategy to prevent the activation of maternal macrophages in a pro-inflammatory profile and avoid tissue damage as described in humans and in murine models [39]–[41].

Regarding chemokine production by monocytes and their migratory properties, Fest et al. showed enhanced chemokine production by monocytes cultured with trophoblast cells stimulated with LPS paralleled by increased monocyte recruitment toward trophoblast cells [16]. This set of experiments was performed with monocytes pre-cultured with trophoblast cells during 24 hours and then challenged with LPS for additional 48 hours. Here, by analyzing chemokine production and migration at 24 hours of co-culture we majorly focused on the first steps of monocyte/macrophage activation once they have interacted with trophoblast and PAMPs stimuli. In fact, after 48 hours we have observed increased monocyte migration as previously reported [16]. On this basis, it is conceivable that restraint in monocyte recruitment would serve as an early strategy, then according to the concentration and nature of the PAMP stimulus this restraint could break and leukocytes would be attracted to the interface with subsequent tissue damage and embryo loss [42].

Another interesting point is that poly [I:C] increased monocyte migration toward Swan-71 cells at 24 h, an effect not seen with LPS or PGN, whereas chemokine expression in monocytes or trophoblast cells was not decreased with poly [I:C] as it was seen with LPS or PGN, suggesting that poly [I:C] has the ability to bypass the initial restraint strategy. The differential effect might be explained by the diverse downstream signaling events and adapter molecules involved in each TLR/ligand system. Accordingly, Koga et al. have reported that poly [I:C] induces preterm delivery in a TLR3-dependent manner in C57B/6 mice [27]. In this model CCL5 production increased 52 times after the stimulation of trophoblast cells with poly [I:C] while CCL2 just 2.5 times, reflecting that each stimulus might differentially modulate chemokine expression.

In humans, chemokine up-regulation within the maternal-placental compartments is associated with microbial invasion and with an exacerbated inflammatory response. As an example, in the chorioamnionitis, placental inflammatory lesions parallel an increase in CXCL10, CXCL11 and CXCL12 concentrations in maternal plasma [42]. Likewise, in the villitis of unknown etiology (VUE), a destructive inflammatory lesion of villous placenta, decidual macrophages appear activated in an inflammatory profile and T helper-1 effector profile is observed [42]. The transcriptome of VUE placentas revealed an increase in a subset of chemokines and their receptors, including CCL5 and CCR5, accompanied by a systemic deregulation of CXC chemokines in maternal and fetal circulation [42]. In the present *in vitro* model, the anti-CCL5 neutralizing Ab significantly reduced the migration of CD14+ cells toward Swan-71 cells stimulated with poly [I:C] highlighting CCL5 contribution for CD14+ cells recruitment.

One of the major findings of the present work is the observation that the modulation of chemoattractant signals upon trophoblast-monocyte interaction is bidirectional: not only monocytes modulate chemokines and their receptor expression in the context of the maternal-placental interaction, but also trophoblast modulate them after culture with monocytes in the presence of PAMPs stimuli.

Therefore, trophoblast cells are able to attract and successfully regulate, ‘educate’, monocytes to produce and secrete a particular set of cytokines and chemokines supporting their growth and survival. Moreover, we propose that at the earliest phases of an infection, trophoblast cells would selectively modulate chemokines and their receptor expression as one of the first steps to control leukocyte trafficking to avoid potential tissue damage. In this sense, Nancy et al. recently reported that decidual stromal cells impaired the accumulation of maternal effector T cells within the decidua by the epigenetic silencing of T cell-attracting inflammatory chemokines genes through repressive histone marks [43].

Finally, regarding the use of trophoblast cell lines as in vitro models, Apps et al. recently analyzed the HLA expression of primary trophoblast cells from normal pregnancies; from the choriocarcinoma cells lines JEG-3 and JAR; and from the placental cell lines HTR-8/SVneo, Swan-71 and TEV-1 [44]. Particularly, for the trophoblast-CD14+ cell interaction focused in this work it is relevant to note that Swan-71 express classical HLA class I molecules but not HLA-G as normal extravillous trophoblast cells. HLA-G is primarily involved in NK rather than monocyte/macrophage interaction with trophoblast cells. Swan-71 cells are a useful in vitro approach to study immune-trophoblast interaction since do not transform in vivo like tumor-derived JEG-3 and JAR cells lines.

In conclusion, among multiple mechanisms operating at the maternal-placental interface for homeostasis maintenance and tolerance induction, trophoblasts appear to ‘instruct’ maternal monocyte/macrophage profile through restraining their migration at early stages of pathological stimulation and this strategy might be selective depending on the PAMPs stimuli.
